# Understanding Water Utilization Mechanisms in Degrading Bamboo Shoots: A Cytological and Physiological Study

**DOI:** 10.3390/plants13141969

**Published:** 2024-07-18

**Authors:** Tianyi Hu, Zhengchun Wu, Meng Deng, Haiwen Liu, Jiao Xiao, Qiang Wei, Fen Yu

**Affiliations:** 1 Jiangxi Provincial Key Laboratory of Improved Variety Breeding and Efficient Utilization of Native Tree Species (2024SSY04093), Jiangxi Agriculture University, Nanchang 330045, China; hutianyi528@163.com (T.H.);; 2 State Key Laboratory of Tree Genetics and Breeding, Co-Innovation Center for Sustainable Forestry in Southern China, Bamboo Research Institute, Key Laboratory of National Forestry and Grassland Administration on Subtropical Forest Biodiversity Conservation, School of Life Sciences, Nanjing Forestry University, Nanjing 210037, China

**Keywords:** *Phyllostachys edulis* ‘Pachyloen’, degrading shoots, parent bamboo, water pressure, water potential, programmed cell death

## Abstract

Degradation of shoots, characterized by stunted growth and signs of water deficit, is common in bamboo stands. However, the specific mechanisms underlying water utilization in degrading shoots remain unclear. This study sought to address this gap by harvesting bamboo shoots and culms of *Phyllostachys edulis* ‘Pachyloen’, employing cytological and physiological techniques to compare water utilization mechanisms between healthy and degrading shoots, and investigating the water supply to bamboo shoots by the parent bamboo. The water pressure in the degrading shoots was markedly lower compared to that of the healthy shoots, and it declined as the degradation progressed, resulting in reduced water content and the cessation of guttation in the degrading shoots. In conditions of water deficit, the percentage of free water in bamboo shoots decreased while the percentages of bound and semi-bound water increased, with the proportion of semi-bound water reaching as high as 88.13% in the late stages of degradation. The water potential of parent bamboo culms of different ages varied at different times of the day and during different growth stages of bamboo shoots, showing a strong association with the development of bamboo shoots. Conversely, the correlation between changes in the water potential of bamboo shoots and their degradation patterns was found to be comparatively minimal. The weakening of the connection between the bamboo shoots and the parent bamboo culms may play a significant role in the degradation of the bamboo shoots. This is evidenced by a decrease in the fluorescence intensity of the nucleus in bamboo shoots and the degradation of genetic material. This study lays the foundation for future research into the mechanisms of bamboo shoot degradation.

## 1. Introduction

Shoot degradation is the process of bamboo shoots slowing down, stopping, and gradually dying during elongation growth, resulting in huge economic losses. It is a common phenomenon in bamboo development and has been reported in several bamboo species [1,2]. However, most of the studies focused on the regulation of shoot degradation, and the related mechanism is still unclear. Previous studies have shown that shoot degradation is a process of competition and adaptation among individual bamboo shoots, a strategy for clonal growth and reproduction in bamboo stands [3], which may be caused by both endogenous (insufficient nutrient supply of bamboo shoots from parent bamboo [4]) and exogenous factors (suffering from pests [5], diseases and mechanical damages, and climatic anomalies [6]). During shoot degradation, the respiration of bamboo shoots is weakened [7], the content of growth-inhibiting substances increases [8], and the content of proteins and non-structural carbohydrates decreases [9,10]. Furthermore, degrading shoots may experience water stress, as evidenced by their morphological features such as the cessation of guttation, withering of the culm sheath, and yellowing of shoots [11], as well as cytological features including cell wall relaxation and silica cell proliferation [12]. These characteristics align with those typically observed in plants under conditions of water deficiency [13].

Water is not only involved in metabolism, reflecting plant growth, but is also a medium for transporting nutrients [14]. Unlike most other plants, bamboo, as a typical clonal plant, has a rhizome system that enables the distribution of water and substances to individual ramets through physiological integration along the water potential gradients, and with more ramets, the physiological integration of water is stronger [15,16]. The parent bamboo can supply water and nutrients to bamboo shoots through physiological integration [17,18]. Before the emergence of bamboo shoots, the water in the rhizomes moves towards the culms, while the most water flows to the bamboo shoots before the leaves function [19,20,21]. Fang found that the removal of mature culms resulted in a significant decrease of 79% in sap flux density rates within the shoots, indicating that the parental bamboo plays a crucial role in supplying the water that is essential for the growth of bamboo shoots [22]. The study further demonstrated that water movement to the base of the bamboo shoot occurs through a combination of root pressure and transpiration pull, with the flow direction being from the bottom to the top of the plant [23,24,25]. Bamboo sheaths play an important role in controlling water transport [26], which in turn impacts the metabolism of bamboo shoots and the absorption of nutrients, particularly non-structural carbohydrates through carbohydrate co-transport [27]. Further investigation into water metabolism within bamboo shoots is crucial for understanding the process of shoot degradation, yet this area of research remains underexplored.

This study comprehensively investigated the water pressure and water status of bamboo shoots during the degrading process, as well as the water potential of parent culms with varying ages in *Phyllostachys edulis* ‘Pachyloen’, a variety of *Phyllostachys edulis* known for its excellent shoot and culm quality. Additionally, the cytological characteristics of the degrading shoots were observed using optical microscopy in conjunction with acridine orange fluorescence staining, and the presence of DNA fragments was detected using a DNA Ladder. This will lay the foundation for elucidating the mechanism of bamboo shoot degradation.

## 2. Results

### 2.1. The Relationship between Guttation and Bamboo Shoot Height Growth

**Figure 1:**
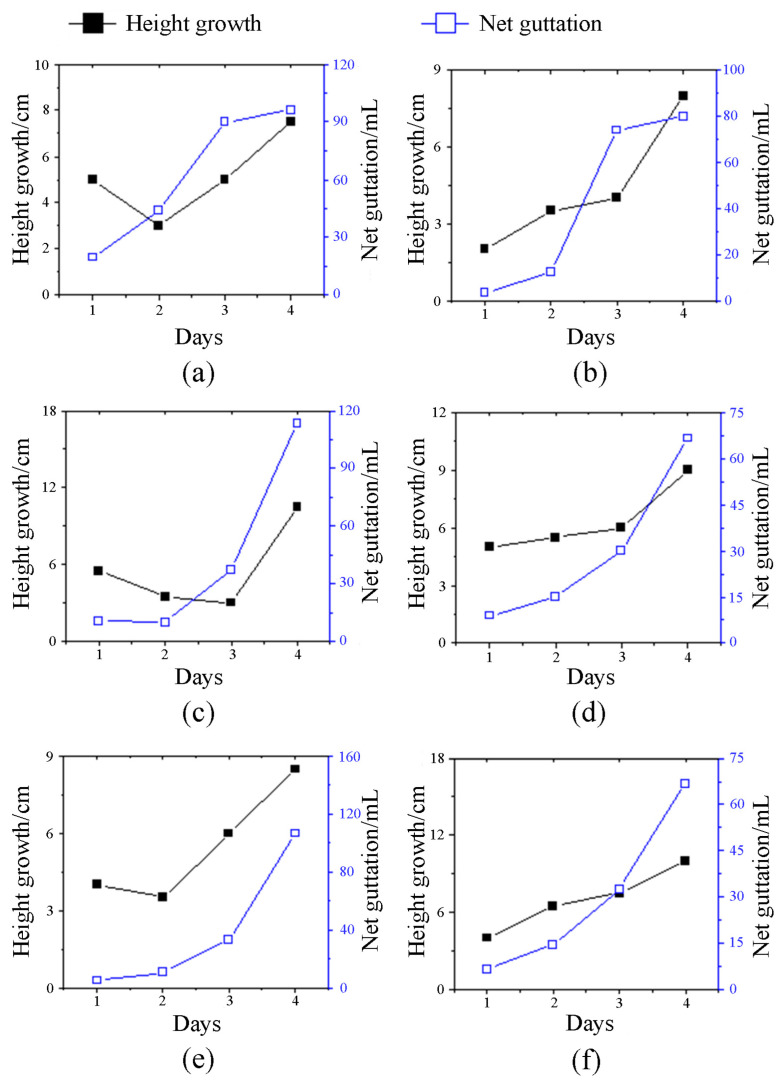
Relationship between guttation volume and the height growth of *Ph. edulis* ‘Pachyloen’ bamboo shoots. (**a**) The first shoot; (**b**) the second shoot; (**c**) the third shoot; (**d**) the fourth shoot; (**e**) the fifth shoot; (**f**) the sixth shoot.

Figure 1 shows that the faster the bamboo shoot grew, the more guttation it produced. A statistical analysis of the relationship between the height growth of bamboo shoots and guttation yielded a correlation coefficient of 0.653, with a significance level of *p* = 0.001 < 0.01. This indicates a significant positive correlation between the two variables.

### 2.2. Water Pressure of Bamboo Shoots during Degradation

**Figure 2:**
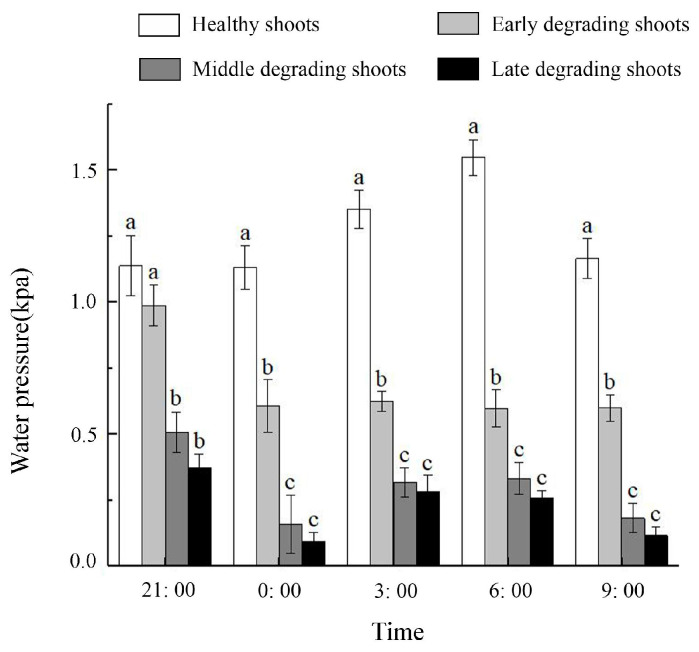
Dynamic change in water pressure at different times in healthy shoots and degrading shoots of *Ph. edulis* ‘Pachyloen’. Different letters in each graph indicate significant differences, *p* < 0.05.

The water pressure of degrading shoots exhibited a gradual decline as they underwent degradation. Degrading shoots and healthy shoots have different patterns of water pressure during nighttime (Figure 2). The water pressure of healthy shoots increased at night and reached a maximum value of 1.55 kpa at 6:00 a.m., and then decreased from 6:00 to 9:00. The water pressure of degrading shoots at night in the early stage firstly declined and then stabilized, with the maximum water pressure of 0.99 kpa at 21:00, while the trend of the water pressure of degrading shoots in the middle and late stages showed an inverted N-shape, with the maximum water pressure of 0.51 kpa and 0.37 kpa at 21:00 and the minimum water pressure of 0.16 kpa and 0.09 kpa at 0:00, respectively. The nighttime water pressure of healthy shoots was always higher than that of degrading shoots.

### 2.3. Water Status of Bamboo Shoots during Degradation

Table 1 illustrates the decline in the water content of bamboo shoots during degradation. Specifically, the water content of early degrading shoots closely resembled that of healthy shoots. However, the water content of middle and late degrading shoots measured 91.84% and 89.31%, respectively, indicating a lower water content compared to healthy shoots. In the healthy shoots, the percentage of free water was the highest at 96.14%, while the proportion of bound water was the lowest at 1.36%. However, in the case of degrading shoots, a comparison with healthy shoots revealed a lower proportion of free water and a higher proportion of semi-bound water. Furthermore, as the bamboo shoots underwent degradation, the proportion of free water in the degrading shoots decreased, while the proportion of semi-bound water increased. Additionally, the proportion of bound water initially increased and then decreased. Semi-bound water accounted for the highest percentage in the late degrading shoots, even up to 88.13%.

### 2.4. Daily Variation in Water Potential of Parent Culms at Different Ages

**Figure 3:**
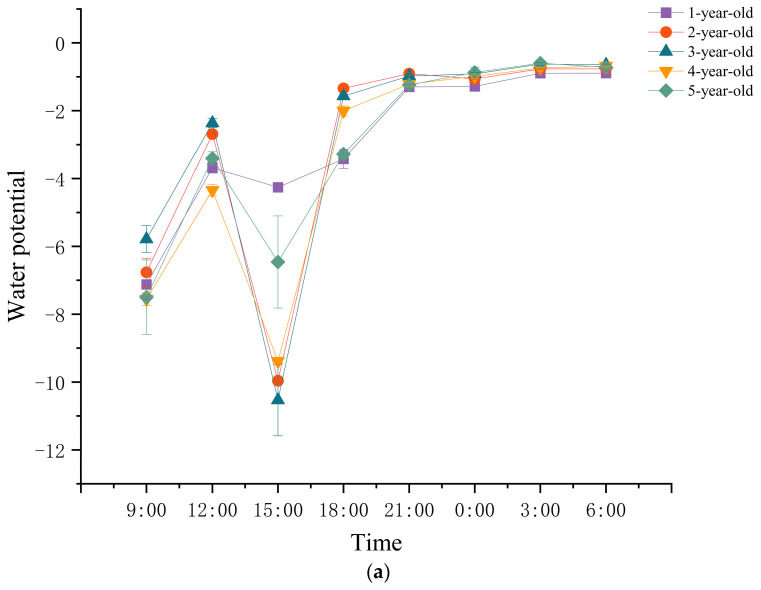

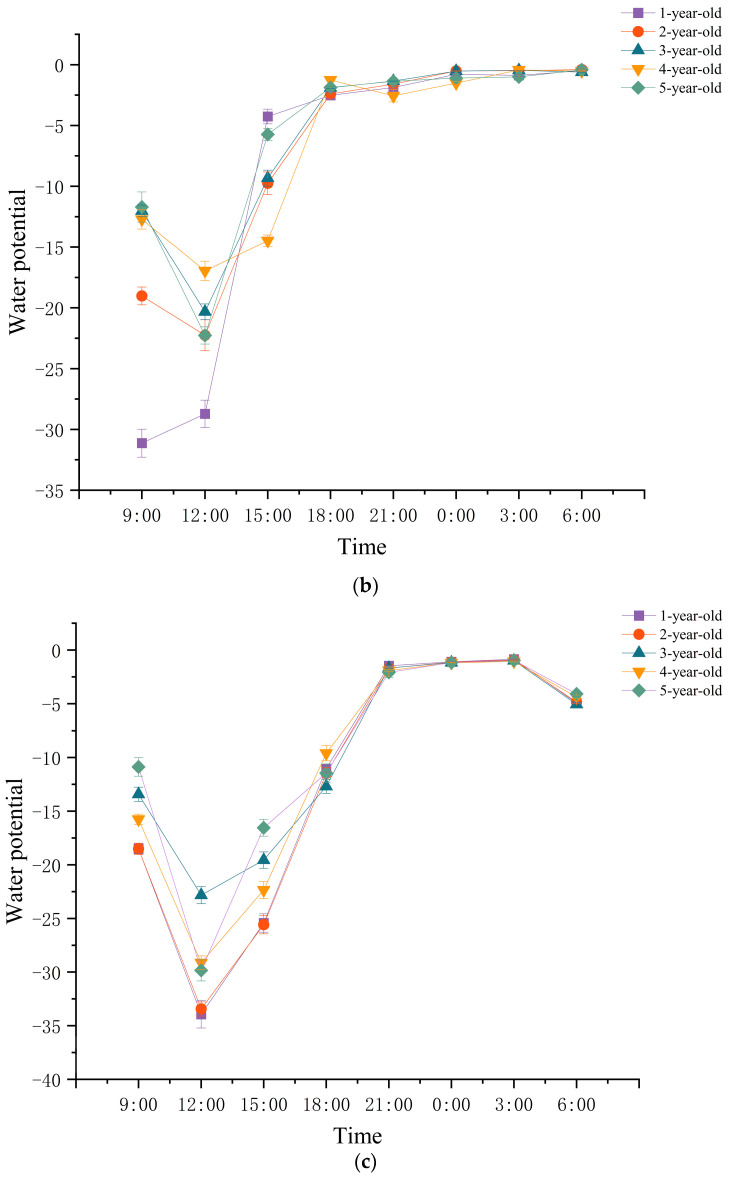
Daily changes in water potential of *Ph. edulis* ‘Pachyloen’ culms at different ages in different stages of bamboo shoot emergence. (**a**) In the early stage of shoot emergence; (**b**) in the exuberant stage of shoot emergence; (**c**) in the last stage of shoot emergence.

The water potential of culms exhibited significant variations across different ages. In general, the water potential of parent culms decreased upon the emergence of bamboo shoots, as depicted in Figure 3. However, during the period of 3:00–6:00, the water potential during the exuberant stage of shoot emergence surpassed that of the early stage. Furthermore, during the final stage of shoot emergence, the water potential reached its lowest point. During a specific time period, the water potential fluctuations in parent culms at various stages of growth exhibited a consistent pattern, characterized by a “double valley” curve during the initial phase of shoot emergence and a “single valley” curve during the subsequent stages of shoot emergence. Furthermore, the water potential of parent culms, regardless of age, reached its peak between the hours of 3:00 and 6:00, while it reached its lowest point between 9:00 and 12:00. Comparing the water potentials of parent culms at different ages, it was found that at the early stage of shoot emergence, the water potentials of 2-year-old and 3-year-old culms were the highest from 9:00 to 21:00, except 15:00. Conversely, the water potentials of 5-year-old culms reached their peak between 0:00 and 3:00. At the later stage of shoot emergence, the water potential of culms aged 4 or 5 years exhibited the highest values between 9:00 and 18:00, while the water potential of culms aged 2 or 3 years was comparatively higher during other time periods. During the final phase of shoot emergence, it was observed that the water potential of 1-year-old bamboo reached its peak between 21:00 and 3:00, while the water potential of 4-year-old and 5-year-old culms exhibited higher levels during the remaining time periods.

### 2.5. DNA Degradation in Bamboo Shoots

**Figure 4:**
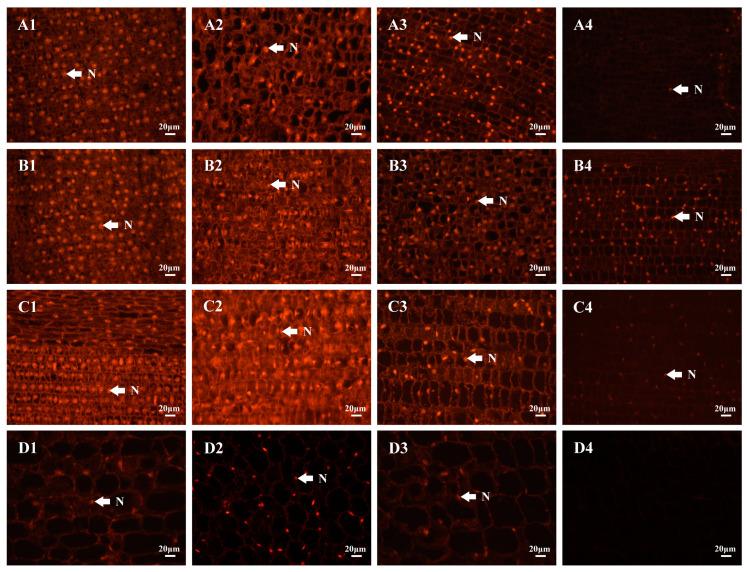
Nucleus fluorescence in healthy shoots and degrading shoots at different developmental stages. (**A1**–**D1**) Healthy bamboo shoots; (**A2**–**D2**) early degrading shoots; (**A3**–**D3**) middle degrading shoots; (**A4**–**D4**) late degrading shoots; (**A1**–**A4**) meristematic stage; (**B1**–**B4**) initial elongation stage; (**C1**–**C4**) rapid elongation stage; (**D1**–**D4**) late elongation stage. N: Nucleus.

In healthy bamboo shoots, the intensity of nucleus fluorescence showed a tendency to increase followed by decreasing with cell development, peaking at 90.76 during the rapid elongation stage and declining to 29.09 during the late elongation stage. Compared to normal shoots, nuclear fluorescence was stronger in early degrading shoots, weaker in middle and late degrading shoots, and weaker during bamboo shoot degradation. During the early and middle stages of degradation, the fluorescence intensity of nuclei at various developmental stages exhibited a pattern consistent with that of healthy shoots, with the highest fluorescence observed during the rapid elongation stage (117.88 and 60.36) and the lowest fluorescence recorded during the late elongation stage (25.92 and 15.74), respectively. In the late degrading shoots, nuclear fluorescence was the strongest at 38.98 at the early stages of elongation and the weakest at 10.89 at the late stages of elongation (Figure 4 and Table 2).

**Figure 5:**
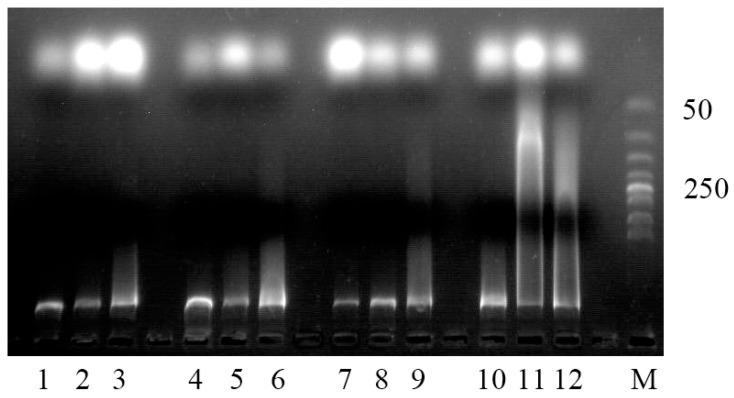
Agarose gel analysis of total DNA isolated from healthy shoots and degrading shoots of *Ph. edulis* ‘Pachyloen’.

Figure 5 illustrates that the electrophoretic results of degrading shoots showed different DNA degradation from that of healthy shoots. Specifically, the electrophoretic results of healthy shoots showed slight trailing only at the base, accompanied by prominent bands in the top and middle regions. Conversely, early and middle degrading shoots exhibit trailing bands in both the middle and base regions, while late degrading shoots present trailing bands at the top and ladder-like bands in the middle and base regions.

## 3. Discussion

### 3.1. Water Pressure and Water Status Were the Key Factors Affecting the Growth of Bamboo Shoots

Guttation is the phenomenon of water leaking from the tips and edges of plant leaves, which is very common in Poaceae plants [28,29,30], and plays an important role in maintaining osmotic pressure [31], enhancing root absorption [32], and preventing pests and diseases [33,34,35]. Plants exhibiting vigorous guttation are indicative of favorable metabolism and growth, while the absence or reduced occurrence of guttation suggests an imbalance in water metabolism, resulting in poor growth or a tendency for senescence [24]. Bamboo also has guttation, and this study found that the faster the bamboo shoots grew, the more guttation they had, so the evaluation of bamboo shoot height growth can be ascertained through the assessment of guttation quantity. The principal constituents of guttation encompassed various compounds, such as organic acids, sugars, and hormones [36]. Our previous research found that the degrading shoots stopped guttation at the early stage, which is the earliest important feature presented by the degrading shoots [8].

Guttation is caused by water pressure in that the higher the water pressure, the more water the plant would exude [24]. Water pressure is related to the uptake and transport of water within the plant [37,38]. Wang concluded that internode elongation was dependent on increased water pressure and elevated soluble sugar content [26]. Notably, the water pressure in typical shoots experiences an augmentation during nighttime hours, reaching its peak at 6:00 a.m. This observation aligns with the established growth pattern of bamboo shoots, which exhibit accelerated growth and water expulsion during nocturnal periods. However, the trend of water pressure in degrading shoots at night was different from that of healthy shoots, which indicated the difference in water utilization between the two. Moreover, reduced water pressure in the degrading shoots hindered water absorption, leading to diminished water content within them.

Water status reflects water utilization in the plant. The degrading shoots exhibited a decrease in the proportion of free water, while an increase was observed in the proportion of bound and semi-bound water, which is associated with enhanced stress tolerance [39,40]. Previous studies showed that the elevated ratio of bound/free water in the plant in the face of drought stress facilitated water retention and enhanced adaptation to the environment [41,42]. The change in the water status of bamboo shoots during degradation might be an adaptive response to water deficit, resulting in a significant shift in the primary role of water towards stress resistance, thereby leading to a substantial reduction in the metabolic rate of the shoots. To make matters worse, due to the collaborative transport of substances and water [27], the lack of water absorption had a direct impact on the access of bamboo shoots to the substances supplied by the parent bamboo through the rhizomes, which led to the depletion of nutrients stored in degrading shoots over the winter months. Chen found more starch degradation and lower soluble sugar content in degrading shoots compared to healthy shoots [10].

The low water pressure and abnormal water status led to a decrease in the physiological metabolism of degrading shoots, and consequently a slowing down of growth until it ceased. Therefore, the water pressure of bamboo shoots was an important factor affecting degradation.

### 3.2. Bamboo Shoot Degradation Was a Self-Regulated Process

Some researchers have considered that the main endogenous cause of shoot degradation is nutrient deficiency, which is due to insufficient supply of parent bamboo. The degradation of bamboo shoots was significantly accelerated and the height of degrading shoots was reduced after the parent bamboo was transplanted. After breaking the rhizomes connecting the bamboo shoots with the parent bamboos, the growth rate of the shoots decelerated, exhibiting typical traits of degradation, including the cessation of guttation, loosened culm sheaths, and yellowed sheath blades [43]. This study observed variations in water supply by parent bamboos in relation to shoot emergence stages and diel rhythm, aligning with the growth characteristics of increased shoot emergence and faster growth at night. However, this pattern differed from shoot degradation. Additionally, parent bamboos of varying ages alternately supplied water during different shoot emergence stages, both diurnally and nocturnally. Previous studies of our team concluded that adult culms and their rhizomes contributed more to the carbon supply of bamboo shoots, and old culms and young and old rhizomes were carbon reservoirs for bamboo shoot development [44]. Thus, the parent bamboo ensure a source of water and nutrients for the growth of bamboo shoots. Moreover, some studies also demonstrated that fertilization did not significantly reduce the rate of shoot degradation [45], indicating that the growth status of the parent bamboo and the supply of water and nutrients affected the growth of bamboo shoots, but they were not the most important causes of shoot degradation, which may be related to the bamboo shoot’s own regulation. Programmed cell death is the active death of cells that is genetically regulated [46,47]. In this study, we found that the cells showed the characteristics of programmed death during bamboo shoot degradation because of the disintegration of the nucleus, the weakening of the fluorescence intensity of the nucleus, and the degradation of the genetic material. Lu’s results showed that DNase activity in early and middle degrading shoots was higher than that in healthy shoots [9]. Bamboo shoot degradation, in which nuclei of degrading shoots condense and degenerate, is a process of plant senescence that is regulated by senescence-related genes [12,48]. The initiation of programmed cell death is a complex process that is influenced by environmental factors and coordinated by a combination of hormones and transcription factors [49,50]. We speculated that bamboo shoots received regulatory signals that weakened their connection with the parent bamboo before shoot degradation, and the bamboo shoots were mainly regulated by themselves during the shoot degradation. However, the main signaling factors received by bamboo shoots and the regulatory network during shoot degradation remain unclear.

## 4. Materials and Methods

### 4.1. Overview of the Experimental Site

The experimental site is located in the Bamboo Garden (28°45′24.1″ N, 115°49′50.2″ E) of Jiangxi Agricultural University, Nanchang, Jiangxi Province, which has a subtropical monsoon climate, with an elevation of 45–50 m. The average annual temperature is 19.58 °C, while the average annual precipitation is between 1600 and 1700 mm. Additionally, the average number of annual sunshine hours is 1772–1845 h, and the soil type is red soil.

### 4.2. Plant Materials

A daily count of *Ph. edulis* ‘Pachyloen’ shoots, measurement of diurnal growth, and collection of guttation from the initial appearance of the first bamboo shoot were conducted. Based on the number of shoots emerged, the bamboo shoot period was divided into three stages: the early stage, the exuberant stage, and the last stage. According Zhao et al. (2021) [12], the degradation process was classified into three stages based on morphological changes and the guttation of bamboo shoots. The selected bamboo culm samples were all healthy and well grown. At least 3 samples were selected.

### 4.3. Guttation Content

More than 30 bamboo shoots were selected for the daily growth measurement and guttation collection in the morning (8:00 a.m.) and at night (10:00 p.m.) using a homemade guttation collection device, and 6 plants were randomly selected for graphical representation.

### 4.4. Water Pressure Determination

A portable manometer (Elecall, Elecall Company, Leging, China) was used to determine the water pressure of healthy shoots and degrading shoots at the early stage, middle stage, and late stage during the exuberant stage of shoot emergence (30 March to 31 March 2021). Measurements were taken every 3 h from 21:00 to 09:00 and repeated 3 times, with the results averaged.

### 4.5. Water Status Determination

The samples of both healthy and degrading shoots at various stages were sent at low temperature to Hangzhou Yanqu Information Technology Co., Ltd. (Hangzhou, China) for analysis of water status using a Niumag low-field nuclear magnetic resonance analyzer (MesoMR23-060H-I).

### 4.6. Water Potential Determination

The water potential of 1–5-year-old bamboo culms was determined in the early stage of shoot emergence (27 February 2021), the exuberant stage of shoot emergence (31 March 2021), and the late stage of shoot emergence (17 April 2021) using a pressure chamber water potential meter (1505D-EXP, PMS Instrument Company, Albany, OR, USA). Measurements were taken every 3 h from 9:00 p.m. to 6:00 p.m. and repeated 3 times, and we took the average.

### 4.7. Observation of Cell Nucleus Fluorescence

Each internode of both healthy shoots and shoots at various stages of degradation, ranging in height from 20 to 30 cm, was collected. The samples were then cut into small pieces measuring approximately 5 mm × 5 mm × 5 mm, fixed in a 50% FAA solution, exhausted with a vacuum pump, embedded in high-efficiency paraffin, and subsequently sectioned using a rotary microtome with a thickness of 8 μm. The sections were stained with acridine orange and examined and photographed under an optical microscope (Zeiss AXIO, Oberkochen, Germany). ZEN 2 (blue edition) software (Version 2.0.0.0, Carl Zeiss Microscopy GmbH, Oberkochen, Germany) was used to make 2.5D plots of the nucleus fluorescence and to count the grayscale values of the fluorescence intensity.

### 4.8. DNA Ladder Observation

After rapid freezing by liquid nitrogen, the samples of the top, middle, and base parts of both healthy shoots and degrading shoots at different stages were stored at −80 °C. The DNA was extracted with a plant DNA kit (Beijing Tsingke Biotech Co., Ltd., Beijing, China), and its purity was evaluated using a nucleic acid protein assay. Gel electrophoresis was performed with 3% agarose, utilizing a 50 bp DNA Ladder from the same supplier as a reference marker.

### 4.9. Statistical Analyses

Experimental data were analyzed by one-way variance analysis (ANOVA) with a Duncan test of significance of difference using SPSS 23.0 software, plotted using Origin 2017 software.

## 5. Conclusions

Reduced water pressure and a decreased proportion of free water are important characteristics of bamboo shoot degradation. Water supply to bamboo shoots from the parent bamboo was not a decisive factor in bamboo shoot degradation. Cells of degrading bamboo shoots were characterized by programmed death. This evidence implicated that the connection between the degrading bamboo shoots and the parent bamboo was weakened, and that the process of degradation was mainly self-regulated by the bamboo shoots themselves. Further investigation will be conducted to explore the molecular mechanism responsible for initiating bamboo shoot degradation. This study provided a reference for further revealing the mechanism of bamboo shoot degradation.

## Figures and Tables

**Table 1:** Proportion of water status of healthy shoots and degrading shoots of *Ph. edulis* ‘Pachyloen’.

	Healthy Shoots	Early Degrading Shoots	Middle Degrading Shoots	Late Degrading Shoots
Water content of bamboo shoots	95.77%	95.82%	91.84%	89.31%
Bound water	1.36% ± 0.00190 a	1.83% ± 0.00346 a	2.68% ± 0.00339 ab	1.07% ± 0.00041 a
Semi-bound water	2.50% ± 0.00551 ab	3.81% ± 0.00740 bc	4.72% ± 0.00116 c	88.13% ± 0.00583 e
Free water	96.14% ± 0.00740 h	94.36% ± 0.01067 g	92.60% ± 0.00323 f	10.79% ± 0.00624 d

Different letters in each graph indicate significant differences, *p* < 0.05.

**Table 2:** Fluorescence intensity during the degradation of *Ph. edulis* ‘Pachyloen’ at different developmental stages.

Developmental Stages	Healthy Shoots	Early Degrading Shoots	Middle Degrading Shoots	Late Degrading Shoots
Meristematic stage	66.98 ± 24.11	68.19 ± 32.41	53.54 ± 28.61	25.31 ± 5.48
Initial elongation stage	76.20 ± 26.47	87.66 ± 28.92	51.99 ± 22.85	38.98 ± 16.55
Rapid elongation stage	90.76 ± 30.37	117.88 ± 31.43	60.36 ± 26.12	34.22 ± 4.86
Late elongation stage	29.09 ± 13.41	25.92 ± 10.98	15.74 ± 4.01	10.89 ± 3.53

## Data Availability

Data supporting the results of this study are available from the authors.
